# Understanding the impact of surface roughness: changing from FTO to ITO to PEN/ITO for flexible perovskite solar cells

**DOI:** 10.1038/s41598-023-33147-6

**Published:** 2023-04-19

**Authors:** Philippe Holzhey, Michael Prettl, Silvia Collavini, Claudiu Mortan, Michael Saliba

**Affiliations:** 1 Adolphe Merkle Institute, University of Fribourg, 1700 Fribourg, Switzerland; 2 Institute for Photovoltaics, University of Stuttgart, Stuttgart, Germany; 3 San Sebastián, Spain; 4 IEK-5 Photovoltaik, Forschungszentrum Jülich GmbH, Jülich, Germany

**Keywords:** Chemistry, Materials science

## Abstract

So far, single-junction flexible PSCs have been lacking in efficiency compared to rigid PSCs. Recently, > 23% have been reported. We therefore focus on understanding the differences between rigid and flexible substrates. One often neglected parameter is the different surface roughness which directly affects the perovskite film formation. Therefore, we adjust the layer thickness of SnO_2_ and the perovskite layers. Furthermore, we introduce a PMMA layer between the perovskite and the hole transporting material (HTM), spiro-MeOTAD, to mitigate shunting pathways. In addition, the multication perovskite Rb_0.02_Cs_0.05_FA_0.77_MA_0.16_Pb(I_0.83_Br_0.17_)_3_ is employed, resulting in stabilized performances of 16% for a flexible ITO substrate and 19% on a rigid ITO substrate.

## Introduction

Stopping climate change demands strong investment and large-scale implementation of renewable energies^1–4^. To ensure that it is important to also explore alternative and novel designs of renewable energies to allow for a widespread usage. One of the most accessible and clean renewable energy sources is Photovoltaic (PV)^5–7^. The majority of PV modules currently are rigid and heavy. An attractive option to further help the spread of PV are flexible solar cells offering to easily deliver energy from otherwise inaccessible areas like leight-weight roofs, walls or even clothes^8^. Further flexible PV offers to lower the balance of system cost especially for residential PV^9^. The main market leader, silicon, is rigid and cannot be used in fully flexible structures. Other PV technologies, like GAAs and CIGS, who can be mounted on fully flexible substrates have faced other challenges like high manufacturing cost or limited efficiencies^10^. A newly emerging material for flexible PV are metal-halide perovskites offering to be an ideal canditate for flexible PV reaching recently the second-highest efficiency for flexible PV after GAAs^11^.

Discovered in 2009, perovskite solar cells had an initial power conversion efficiency (PCE) of 3.9%. Now, more than 10 years later, they are rapidly approaching established PV technologies such as silicon having certified efficiencies of 25.7%^12–14^. In a silicon/perovskite tandem they even exceed the single-junction efficiency record of silicon of 26.7%, achieving a certified efficiency of more than 31.3%^15^. Lead halide perovskites have exceptional material properties, including a sharp absorption edge^16^, solution processability^17^, and a tunable bandgap from 1.2 to 2.3 eV^18–20^ by interchanging the above cations, metals, or halides. The potential applications of PSCs range from residential PV, flexible wearable devices, and low-sun intensity applications for the internet of things to residential PV systems^9,21–23^.

Flexible PSCs have been lacking in efficiency compared to rigid ones with the highest reported efficiencies of 23.6%^24^ compared to 25.7%^25^. To understand this further, we try to understand the difference between the most commonly used highly efficient, conventional, thick and rigid FTO substrates to ITO, at first on rigid and then flexible PET/ITO substrates. We have to change to ITO as the flexible polyethylene naphthalate (PEN) substrate is not resistant to high temperatures which are necessary for the fabrication of FTO. Here, we use a low temperature processed planar device architecture (FTO/SnO_2_/perovskite/spiro-OMeTAD/Au), which reached PCEs > 20% in the past on a rigid FTO and transfer it on ITO substrates^26^. In addition, a modified multication perovskite composition was used, where the precursor can be formulated as Rb_0.02_Cs_0.05_FA_0.77_MA_0.16_Pb(I_0.83_Br_0.17_)_3_ (from here on simply “RbCsMAFA”)^27^.

We identify three areas precluding rigid PSCs with FTO from the transfer to flexible ITO substrates. First, the electron transport material layer, SnO_2_, requires a different layer thickness. Secondly, the perovskite layer thickness itself needs changing and thirdly a thin interfacial PMMA layer is introduced to prevent shunting pathways between the HTM, spiro-OMeTAD, and the ETM, SnO_2_. Following these steps, rigid ITO devices were achieved with a stabilized PCE of 19.1% and flexible substrates with a stabilized PCE of 16%. We posit that one of the main challenges for achieving highly-efficient flexible perovskite solar is the different surface roughness.

## Towards efficient, flexible devices–the differences between ITO and FTO as transparent conductive oxides for perovskite solar cells

Most rigid PSCs have been processed on FTO, which is unsuitable for flexible PSCs as it requires a high processing temperature when manufactured. It is, therefore, necessary to change from FTO to ITO. The two materials have different Fermi levels due to their different dopants, influencing the energy band level alignments to the other materials of the solar cell stack. For this work, however, the highest open-circuit voltage (V_oc_) is at 1.18 V for ITO, which is comparable to some of the highest reported V_oc_ for FTO and SnO_2_ 1.21 V^26^. The energy band alignment levels depend on several factors, such as the work function, the substrate treatment, deposition method, and interfaces, involving the possible occurrence of interface dipoles^28,29^. The discussion about energy band alignments in perovskites often does not consider the critical relationship between surface roughness and subsequently altered film formation. Following this, we further characterized the surfaces in terms of roughness and contact angle (see Table 1). Our hypothesis is that the smoother surface of ITO influence the perovskite film formation significantly, leading to more regular, block-like grain boundaries.

**Table 1:** Surface roughness and contact angle of perovskite solution on ITO, polished FTO, and FTO.

	R_q_	R_a_	Contact angle
ITO	2.154 nm	1.765 nm	67.8° ± 1.24°
Polished FTO	14.963 nm	11.275 nm	58.0° ± 1.19°
FTO	45.425 nm	35.916 nm	52.0° ± 1.9°

The root mean squared of the vertical deviations of the roughness profile from the mean line is R_q_ and the arithmetic average of the vertical deviations is R_a_. The values have been quantified through atomic force microscopy (AFM) measurements.

**Figure 1:**
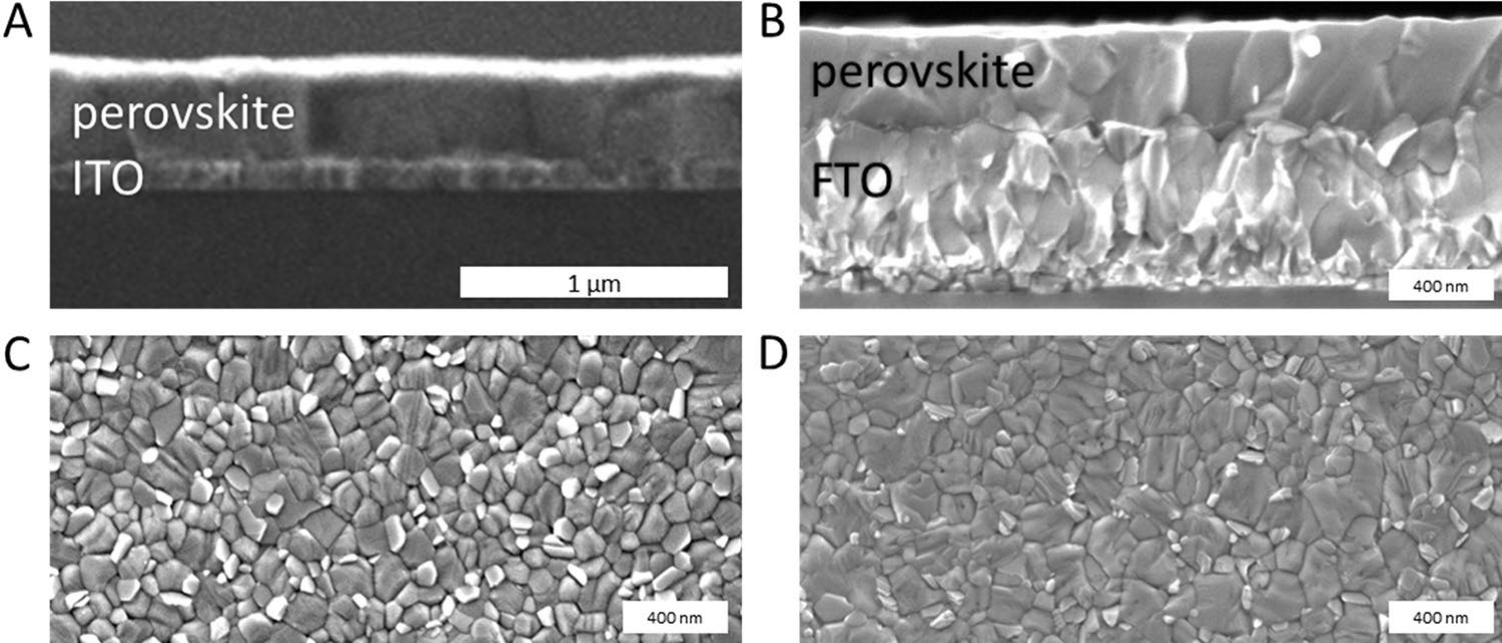
SEM of the perovskite layer on substrates with different surface roughness. The grain size form of the perovskites correlates with the surface roughness of the substrate. (**A**) Perovskite on a smooth ITO substrate showing relative straight grains. (**B**) Perovskite on SnO_2_/FTO, the roughest substrate, with ca. 48 nm surface roughness showing less orientation orthogonal to the substrate. (**C**) Top-view SEM image of perovskites layers on ITO and on (**D**) FTO. Voids between crystals are more clearly pronounced on the ITO substrate.

The measured contact angle for a polished FTO substrate is 58.0° ± 1.19°, which lies between the surface roughness of FTO and ITO. This is consistent with the trend of better wettability, correlating with increasing surface roughness for contact angle below 90°^30^. Cross-section and top-view scanning electron microscopy (SEM) images of ITO resp. FTO with a 15 nm SnO_2_ layer and a perovskite layer are shown in Fig. 1. The rough FTO substrate has a perovskite layer with numerous, ragged grain boundaries, and other irregularities, seeming to have a less ordered orientation. In contrast, the crystals on the smooth ITO substrate are more distinct and block-like. The grain boundaries tend to go from the bottom to the top contact. We checked our hypothesis on a polished FTO substrate, which has less surface roughness. In Fig. S1, we observed more similar grain behavior in line with the smooth ITO surface. Thus, there is a trend that a smoother surface correlates with more regular, block-like grain boundaries.

Although all fabrication parameters and the precursor solution were the same for all devices, the perovskite layer thickness on the ITO is only 340 nm which is about 2/3 of the layer thickness on FTO. We measured the contact angle of the perovskite precursor on ITO, polished FTO and FTO (all with a SnO_2_ compact layer), showing that the wettability correlates with surface roughness (see Table 2), which resulted in a thinner perovskite thickness. These thinner layers harvest less incoming light, and consequently, full devices on ITO showed a lower current density of 19–20 mA cm^−2^ compared to FTO with around 22 mA cm^−2^. Furthermore, the ratio of shunted solar cells occurred more frequently for smooth ITO than for rough FTO, with a reduced fill factor (FF) of 10% for ITO compared to FTO. These observations can be linked to the SEM images (Fig. 1): ITO shows a higher abundance of cracks due to the more monolithic crystal growth that also may lead to a thinner perovskite layer (Fig. 1A,C) compared to the ones on FTO (Fig. 1B,D). Lowering the shunt resistance resulting in a decreased fill factor which is more likely to occur on smooth ITO. Based on these observations, we use three optimization steps to tackle the different substrate properties to reach higher efficient devices:

**Table 2:** Overview of the best device characteristics on rigid and flexible ITO substrates.

	V_OC_ [mV]	J_SC_ [mA cm^−2^]	FF	PCE [%]	Stabilized PCE [%]
Rigid ITO	1183	21.17	0.74	18.4	19.1
Flexible ITO	1128	19.74	0.69	15.7	16.0

**Figure 2:**
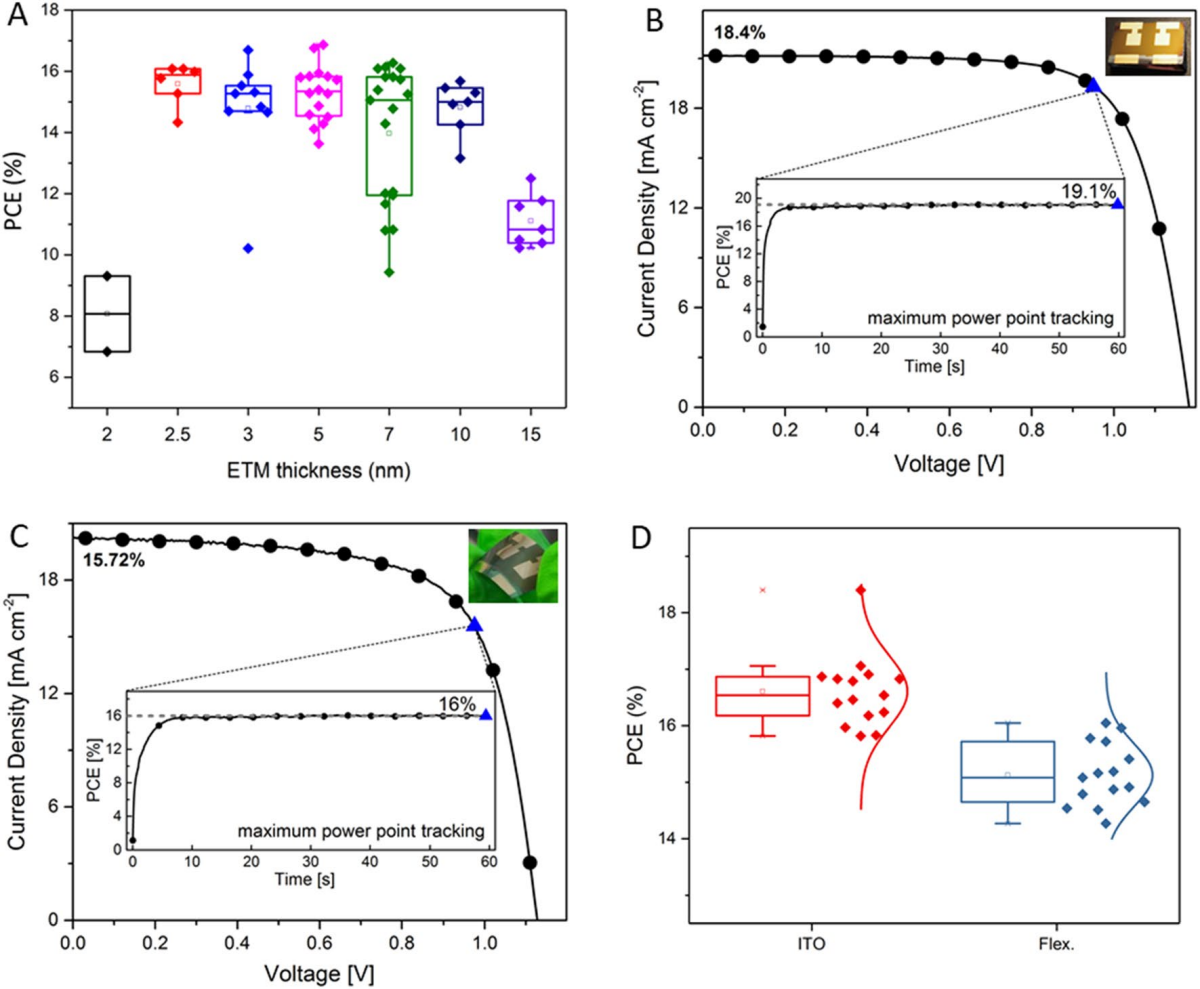
(**A**) Thickness optimization of the SnO_2_ layer with at least 5 devices per thickness from 0 to 15 nm. We observe a decrease of reproducibility (out of 10 devices only 2 were working) for layer thicknesses less than 5 nm. (**B**) J-V curve of the best rigid cell on ITO with an additional thin PMMA layer between the perovskite and HTM. A stabilized PCE of 19.1% was achieved and 18.4% in the J-V scan. (**C**) J-V curve of the best flexible solar cell on PET/ITO with an additional thin PMMA layer. A stabilized PCE of 16.0% was achieved in the MPP measurement and 15.7% in the J-V scan. (**D**) Solar cell characteristics of 15 cells made on rigid and flexible ITO substrates.

Firstly, the smoother surface of the ITO allows for a thinner SnO_2_ layer which improves the charge carrier extraction as shown by Stolterfoht et al. for PTAA^31^. In general, considerably thicker layers are required to ensure a pinhole-free layer due to the roughness of the transparent conductive oxide (TCO). With a smoother surface, the layer thickness can be reduced without risking pinholes that increase the risk of shunting. Samples with a SnO_2_ thickness of 2 to 10 nm and with 15 nm (the current standard for the FTO reference) were fabricated. As shown in Fig. 2A, a slight performance improvement could be achieved down to 5 nm without reducing reproducibility. Below 5 nm more devices get shunted as the SnO_2_ layer does not fully cover the ITO electrode anymore. Since the reproducibility is higher, and the average efficiency is not significantly lower, we decided to use 5 nm as the optimal thickness for the planar architecture on ITO. Lower thickness can achieve higher efficiencies but also reduces reproducibility. We hypothesize, as observed by Stolterfoht et al.^31^, that the thinner charge transport layer leads to a faster charge extraction and thus to a lower recombination rate with higher performance.

The second optimization targets the decreased current density observed for the different wettability of the ITO substrate compared to the FTO substrates. We observed no difference between the flexible and rigid substrates. To increase the short circuit current density (J_SC_), we increase the layer thickness by lowering the maximum spin speed during the perovskite deposition. The correlation of the layer thickness as a function of spin speed is shown in Fig. S2. The J-V curves show an improved current density by ca. 1–2 mA cm^−2^ without decreasing the reproducibility.

The third optimization approach targets the voids between the perovskite crystals and the consequent shunting. It is likely that the conductive spiro-OMeTAD penetrates through the voids to directly contact the ETL. Therefore, a thin isolating buffer layer between the perovskite layer and the spiro-OMeTAD could prevent such a shunting pathway. Thus, a thin layer of Poly(methyl methacrylate) PMMA, using a 0.1 mg/ml solution in chlorobenzene (CB), is deposited on top of the perovskite layer before the HTM. The introduction of an additional PMMA layer reduces the fraction of shunted devices from about 80% to 0%. We hypothesize that since we spin-coat spiro-OMeTAD, dissolved in CB, on top of the PMMA layer, the HTM solution may dissolve the PMMA on the surface but not the PMMA penetrated within the voids in the perovskite layer. This seems likely as we observe no difference in the J_SC_ (20.3 ± 0.6 mA cm^−2^ for the control, compared to 20.5 ± 0.3 mA cm^−2^ for the PMMA devices), V_OC_ (1.13 ± 0.02 V for the control, compared to 1.13 ± 0.02 V for the PMMA devices), and FF (0.68 ± 0.04 compared to 0.68 ± 0.05).

Finally, we use an architecture with 5 nm of SnO_2_, 480 nm of RbCsMAFA perovskite with PMMA on top, followed by spiro-OMeTAD. All three optimizations (SnO_2_ and perovskite layer thickness adjustment, additional PMMA layer) together lead to a PSC on a rigid ITO substrate with a stabilized PCE of 19.1% as shown in Fig. 2B. We used the same procedure to transfer this architecture onto flexible substrates with PET/ITO. Without any further modification, it was possible to use the previously used optimization for rigid ITO. We achieved up to 16% stabilized power output as shown in Fig. 2C. The efficiencies of 15 devices on rigid and flexible ITO substrates are shown in Fig. 2D (all parameters are shown in Fig. S3).

Our fully optimized rigid PSCs show efficiency improvements to the original parameters with FTO from 6 to 7% (absolute), from 12% to a stabilized PCE of 19.1%. The most significant improvement however, is the increase in the reproducibility of the cells. The fraction of shunted devices decreased drastically from about 80% to 0% in the fully optimized architecture. Considering that ITO has a different work function than FTO, the V_OC_ remains high at 1.18 V (Table 2). The parameter which stays relatively low and hampers the cell from going towards 20% efficiency and beyond is the J_SC_. The highest J_SC_ achieved so far in this work was only 21.17 mA cm^−2^. Planar cells on FTO reached a J_SC_ close to 26 mA cm^−2^^32^. The PCE of the solar cells fabricated on the flexible ITO substrate reached efficiencies of 16%, which is 3.1% less efficient than on the ITO substrate. The reduction in efficiency originates from the low fill factor of 0.69 and the lower J_SC_ of 19.7 mA cm^−2^. The variances might stem from further differences between the rigid and flexible ITO substrates, like macroscopic substrate planarity.

## Conclusion

We provide guidelines on transferring from conventional but rough FTO, which is not compatible with a flexible substrate, to smooth ITO. Surface roughness emerges as highly important, affecting the perovskite growth drastically, with the smooth substrate (ITO) exhibiting more monolithic film formation. Following this, three optimization approaches were implemented, such as a reduction of the SnO_2_ layer thickness from 15 to 5 nm, an increase in perovskite film thickness through a lowered spin speed, and a thin PMMA buffer layer. This results in PSCs with a stabilized efficiency of 19.1% on a rigid ITO substrate and stabilized 16% on a flexible ITO/PET substrate. This optimization work on ITO is an important step towards the commercialization of flexible PSCs and gives general guidelines on transferring optimized perovskite architecture from FTO to ITO.

## Supplementary Information

Supplementary Information.

## Data Availability

The datasets used and/or analysed during the current study available from the corresponding author.
