# Supplementary material for: Understanding the impact of surface roughness: changing from FTO to ITO to PEN/ITO for flexible perovskite solar cells
Source: Sci Rep. 2023 Apr 19;13:6375. doi: 10.1038/s41598-023-33147-6 (PMC10115802; doi:10.1038/s41598-023-33147-6)
Supplement: Supplementary file 1 — Supplementary Information. [file 41598_2023_33147_MOESM1_ESM.docx]

Supplementary Information

**Fabrication of perovskite solar cells**

Preparation and cleaning of substrates

The FTO glass was etched with a 4% HCl-solution. Next, the glass was immersed for 15 min in a piranha solution, which was prepared by mixing 2/3 of sulfuric acid (H_2_SO_4_) with 1/3 hydrogen peroxide (H_2_O_2_). The solution was removed, and the substrates were rinsed with deionized water and dried with an air gun. The ITO glass was cut into pieces of 48 x 98 mm^2^. Firstly, the ITO was brushed on all surfaces by using a 10% Hellmanex water solution. Then cleaned by sonication in 2% Hellmanex water solution for 15 minutes. After rinsing with deionized water, the step was repeated with an ethanol solution. Finally, the glass was rinsed with deionized water and rubbed carefully with a glove. The PET/ITO foil sheets were etched with a 4% HCl-solution and only cleaned with deionized water. Then all substrates were further cleaned with a UV ozone treatment for 15 minutes.

Fabrication of the electron selective layer

The electron selective layer was deposited through atomic layer deposition (ALD). The specialty of the ALD process is that monolithic layers of a material can be formed. Tetrakis(dimethylamino)tin(IV) (TDMASn, 99.99%-Sn, Strem Chemicals INC was heated at 65 °C. The substrates were put in the ALD chamber which was preheated to 100 °C.

First, the chamber is flushed with the first reactant TDMASn, then as second reactant ozone is gushed into the chamber. The ozone reacts with the TDMASn precursor and forms SnO_2_ molecules on the surface of the substrates. Third, the chamber is evacuated after a waiting time of approximately 15 seconds. The growth rate per cycle was 0.065 nm and was measured with ellipsometry.

Preparation of the perovskite precursor

The organic cation halide salts, FAI and MABr were purchased from Greatcell (former Dyesol); the lead compounds PbI_2_ and PbBr_2_ from TCI and CsI and RbI from abcr GmbH.

The MAFA perovskite precursor solutions were deposited from a precursor solution containing FAI (1 M), PbI_2_ (1.1 M), MABr (0.2 M) and PbBr_2_ (0.2 M) in anhydrous DMF: DMSO 4:1 (v:v). This composition contains a lead excess as reported elsewhere.^26^ The CsI (1.5 M) solution was dissolved in DMSO. The RbI (1.5 M) was dissolved in DMF: DMSO 4:1.

The CsI and MAFA solution were mixed in a volume ratio of 5:95 to get the CsMAFA solution. The RbI solution was then mixed with the CsMAFA solution in a volume ratio of 2:98.

Deposition

First, the substrates were treated in a UV-ozone for 15 min to clean the surface and to remove residual organic substances. After placing the substrates into a glovebox filled with nitrogen, the substrates were heated for 5 min to 100 °C to remove residual water, for example from a condensate of atmospheric water vapor and were cooled to room temperature prior to the deposition. Furthermore, a temperature of 24-27 °C in the glovebox proved to be beneficial for the deposition and crystallization of the perovskite. The deposition of the perovskite can be divided into three steps. The first step is to deposit the perovskite precursor on the glass substrate. The substrates were placed in the spin coater and 40 µl of the precursor was dropped in the middle of the glass substrate. For the planar devices, it was important to spread the perovskite precursor evenly over the surface of the substrate with the tip of the pipette. Hereafter the following spin coating program was started:

|  | Duration | Speed | Ramp |
| --- | --- | --- | --- |
| Step 1 | 10 s | 1000 rpm | 200 rpm/s |
| Step 2 | 20 s | 3000 rpm - 6000 rpm | 1000 rpm/s |

Five seconds before the end of the spin coating program, 200 µl chlorobenzene (CB) were dropped on the substrates. The most critical factor for a good crystallization is the way that the antisolvent is dropped. A “smooth” dropping with a steady flow of 0.5 to 1 s turned out to result in the best device performances. Directly after the spin coating, the substrates were dried and annealed on the hotplate at 100 °C for 60 min.

PMMA, HTM and the top electrode

In the first step of the preparation, the spiro-OMeTAD powder is dissolved in the solvent chlorobenzene (CB). The ratio is 1.094 ml of CB per 100 mg of Spiro-OMeTAD powder. The solution is heated to 60 °C for about 2 min, shaken and left to cool down for 5 min. The following dopants are added to 100 mg spiro-OMeTAD:

- 39 µl of 4-tert-butylpyridine (TBP),
- 23 µl of bis(trifluoromethylsulfonyl)imide lithium salt (Li-TFSI) of a 1.8M acetonitrile solution.
- 10 µl of tris(2-(1H-pyrazol-1-yl)-4-tert-butylpyridine)-cobalt(III) tris(bis(trifluoromethylsulfonyl)imide) (FK209) of a 0.25 M acetonitrile solution.

The dopants have a molar ratio of 3.3, 0.5 and 0.03 respectively. For the PMMA layer 0.1 mg mL^-1^ PMMA powder was dissolved in CB. 50 µl of the PMMA solution was applied on the perovskite and spin-coated at 4000 rpm for 20s with a ramp of 2000 rpm s^-1^. After ten minutes storage in the nitrogen glovebox, the spiro-OMeTAD solution was deposited. 50 µl of the spiro-OMeTAD solution was dropped on the substrate shortly after starting the spin coating program (dynamic spin coating). It was important to drop the solution with a fast push of the pipette. Finally, 80 nm gold was evaporated as the top contact.

**Measurement techniques**

Solar cell characterization

The characterization of the solar cells was conducted with Class AAA solar simulator (Newport VeraSol-2 LED Class AAA Solar Simulator) and a variable voltage source with a built-in amperemeter (Keithley 2400). The intensity of the light was calibrated with a silicon photodiode, equipped with an IR-cutoff filter (KG3, Schott) and set to 1000 W/m² according to AM1.5 reporting conditions. The cells were mounted into a fixed holder with a black metal mask to fix the active area to 0.16 cm² and to reduce the effects of scattered light. All devices were measured with a scan rate of 10 mV s^-1^ in forward (1.3 V to 0 V) and in the backward scan direction (0 V to 1.3 V). The cells were not preconditioned by light soaking or extended forward biasing in dark. No anti-reflecting coating was used for any cells. For the maximum power point measurements, a JV-curve was measured. The bias voltage was set to the initial maximum power point (MPP). The tracking of the MPP was conducted through a standard perturb and observe method, which constantly adapts the MPP.

Scanning Electron Microscopy

SEM was performed on a ZEISS Merlin HR-SEM.

Atomic force microscopy

Atomic force microscopy was performed on a Digital Instruments Nanoscope IIIa - microscope.

Contact angle measurement by drop shape analyzer

The measurement of the contact angle was performed by the drop shape analysis method with the Krüss Drop Shape Analyzer DSA 100. For correct measurement of the contact angle through this method, it is important to determine the maximum drop size before the drop starts spreading over the surface. The maximum drop size was determined by steadily increasing the drop size with the automatic dispenser of the DSA 100.

After determining the maximal drop size, the contact angle is automatically determined by the program of the DSA 100 from a lateral image of the drop and surface. The contact angle measurement was performed for water and perovskite precursor on different substrates and always on the layer on which it would be deposited.

###### Supplementary Figures


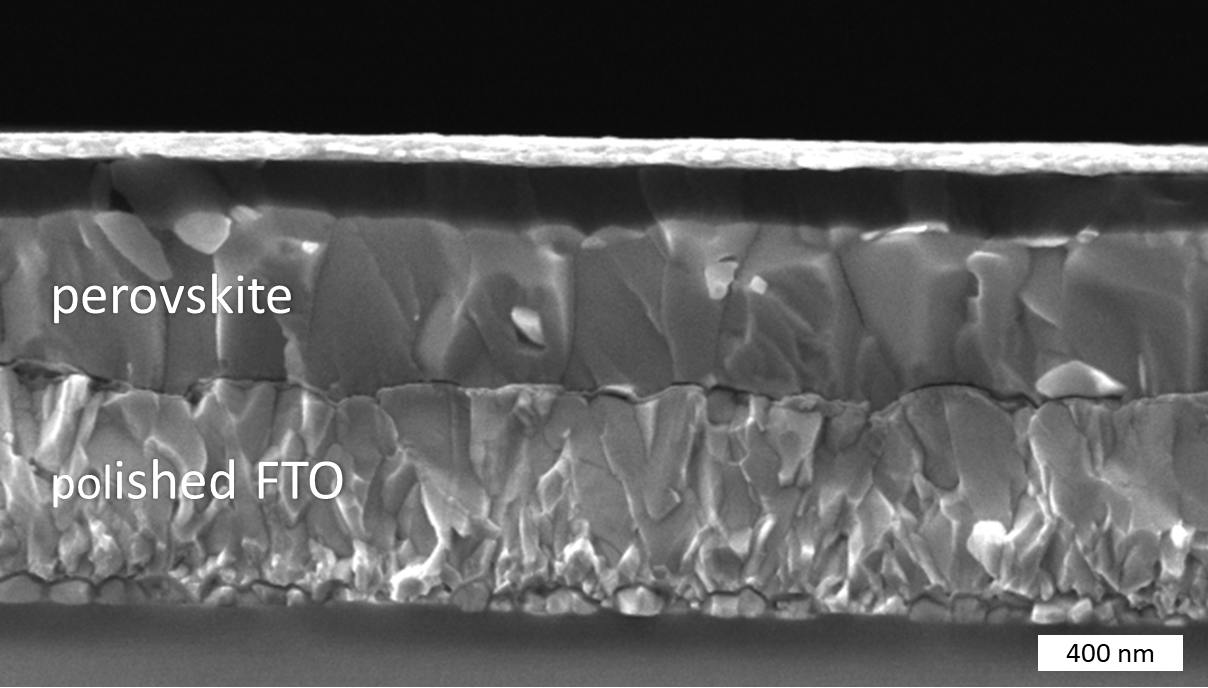


**Figure S1** SEM of polished FTO with a 15 nm SnO_2_ layer and a perovskite layer on top. The SEM shows that the surface structure of the FTO substrate determines the monolithic of the perovskite on top.


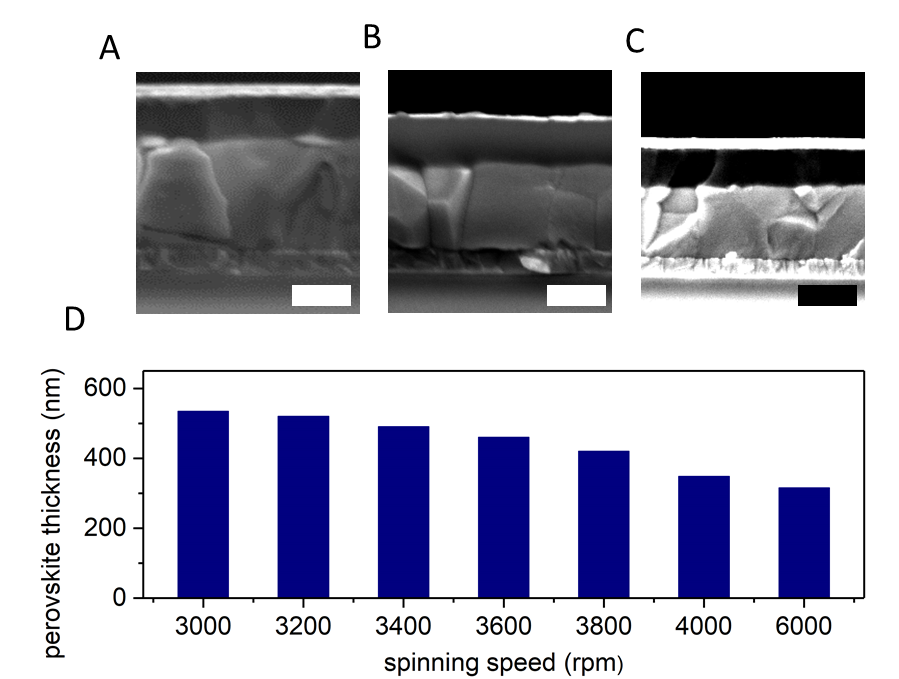


**Figure S2** SEM image of PSC with different spin speeds of the perovskite precursor. Scale bar is 200 nm (**B**) 3000 rpm, (**C**) 4000 rpm, (**D**) 6000 rpm. (**E**) Perovskite layer thickness measured by SEM vs. the maximum spin speed during deposition.


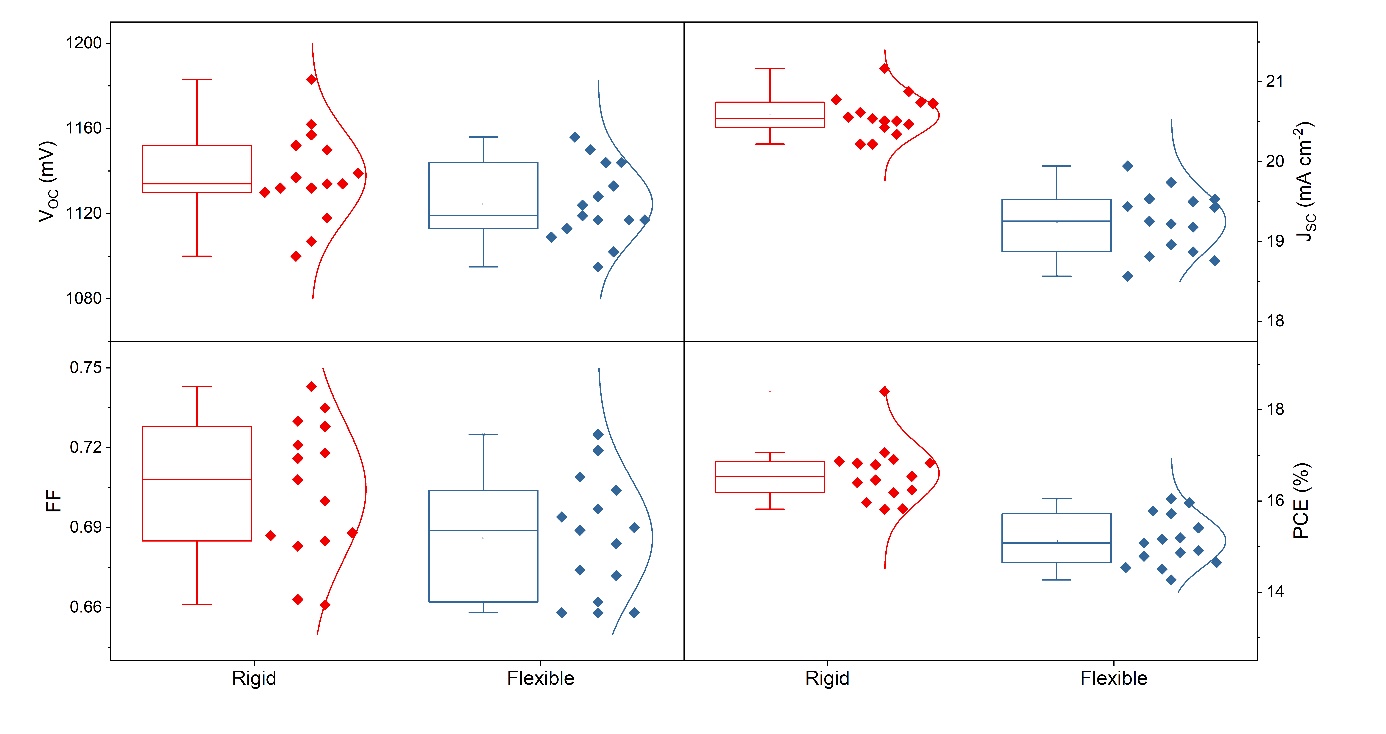


**Figure S3** Photovoltaic parameter of 15 devices on rigid and flexible ITO substrates as shown in Figure 3D.
